# Unique N-terminal sequences in two Runx1 isoforms are dispensable for Runx1 function

**DOI:** 10.1186/s12861-017-0156-y

**Published:** 2017-10-18

**Authors:** Sebastian Nieke, Nighat Yasmin, Kiyokazu Kakugawa, Tomomasa Yokomizo, Sawako Muroi, Ichiro Taniuchi

**Affiliations:** 1 Laboratory for Transcriptional Regulation, RIKEN Center for Integrative Medical Sciences (IMS). 1-7-22 Suehiro-cho, Tsurumi-ku, Yokohama, 230-0045 Japan; 2 Abteilung Immunologie, Interfakultaeres Institute fuer Zellbiologie, Auf der Morgenstelle 15, 72076 Tuebingen, Germany; 3 Laboratory for Immune Crosstalk, RIKEN Center for Integrative Medical Sciences (IMS), 1-7-22 Suehiro-cho, Tsurumi-ku, Yokohama, 230-0045 Japan; 4 Cancer Science Institute of Singapore, National University of Singapore, 14 Medical Drive, #12-01, Singapore, 117599 Singapore; 5 Faculty of Life Sciences (Microbiology), University of Central Punjab, 1 - Khayaban-e-Jinnah Road, Johar Town, Pakistan; 6 International Research Center for Medical Sciences, Kumamoto University, 2-2-1 Honjo, Chuo-ku, Kumamoto City, 860-0811 Japan

**Keywords:** Runx1 proteins, Promoters, Isoform, Hematopoiesis, Translational start site

## Abstract

**Background:**

The Runt-related transcription factors (Runx) are a family of evolutionarily conserved transcriptional regulators that play multiple roles in the developmental control of various cell types. Among the three mammalian Runx proteins, Runx1 is essential for definitive hematopoiesis and its dysfunction leads to human leukemogenesis. There are two promoters, distal (P1) and proximal (P2), in the *Runx1* gene, which produce two Runx1 isoforms with distinct N-terminal amino acid sequences, P1-Runx1 and P2-Runx1. However, it remains unclear whether P2-Runx specific N-terminal sequence have any specific function for Runx1 protein.

**Results:**

To address the function of the P2-Runx1 isoform, we established novel mutant mouse models in which the translational initiation AUG (+1) codon for P2-Runx1 isoform was modulated. We found that a truncated P2-Runx1 isoform is translated from a downstream non-canonical AUG codon. Importantly, the truncated P2-Runx1 isoform is sufficient to support primary hematopoiesis, even in the absence of the P1-Runx1 isoform. Furthermore, the truncated P2-Runx1 isoform was able to restore defect in basophil development caused by loss of the P1-Runx1 isoform. The truncated P2-Runx1 isoform was more stable than the canonical P2-Runx1 isoform.

**Conclusions:**

Our results demonstrate that the N-terminal sequences specific for P2-Runx1 are dispensable for Runx1 function, and likely serve as a de-stabilization module to regulate Runx1 production.

**Electronic supplementary material:**

The online version of this article (10.1186/s12861-017-0156-y) contains supplementary material, which is available to authorized users.

## Background

Runx transcription factor complexes are evolutionarily conserved heterodimers that consist of an α-subunit, referred to as the Runx protein, and a non-DNA binding β-subunit. Runx complexes recognize a specific DNA sequence (5′-PyGPyGGT-3′) through their conserved Runt-domain [1–3]. In mammals, three genes encode three distinct Runx proteins, Runx1, Runx2 and Runx3. Ablation or attenuation of each *Runx* gene function in several species revealed that Runx complexes play pivotal roles in the development of many cell types [2, 4, 5]. For example, Runx1 is required for definitive hematopoiesis in vertebrates. Genetic ablation of *Runx1* in mice blocks hematopoietic stem cell (HSC) generation and results in embryonic lethality at approximately 12.5 days post-coitum (dpc) and hemorrhages in the central nervous system (CNS) [6, 7]. Runx1 has also been implicated in human leukemia [8]. Generation of fusion proteins such as RUNX1/ETO and RUNX1/Evi1 through leukemic associated-chromosomal translocation are frequently observed in acute myeloid leukemia (AML) [9]. In addition, mutations in the *RUNX1* gene have been observed in a significant fraction of AML patients [10]. Thus, understanding of how Runx1 expression and function are regulated is fundamental to the field of hematology.

All mammalian Runx genes are transcribed from distal (P1) and proximal (P2) promoters [3]. The *P1-Runx1* promoter is located 130 kb upstream of the *P2-Runx1* promoter in the murine *Runx1* locus [11]. The 5′ untranslated regions (UTR) is short in the *P1-Runx1* transcript, whereas in *P2-Runx1* transcript, it spans more than 1.6 kb and contains GC-rich regions [11] and a putative internal ribosomal re-entry (IRES) element upstream of the translation initiation AUG (+1) [12]. Thus, atypical cap-independent and IRES-dependent translation mechanisms are involved in the translation of *P2-Runx1* transcripts. In addition to these differences in translational control, the expression patterns of *P1- and P2-Runx1* transcripts are also different [13–15]. Expression of the *P2-Runx1* transcript is detected earlier in mouse embryogenesis than the *P1-Runx1* transcript [13, 16, 17]. In addition, the products of *P1-* and *P2-Runx1* transcripts, hereafter referred to as P1-Runx1 and P2-Runx1 proteins, have distinct N-terminal sequences [3, 13]; ASDSIFESFPSYPQCFMR and RIPV are unique within the N-terminus of P1-Runx1 and P2-Runx1 proteins, respectively. These isoforms were previously shown to be different in terms of their effect of exogenous expression on cell growth and differentiation of myeloid progenitor cell line [13]. This suggests that distinct N-terminal sequences could confer different functionality to the P1-and P2-Runx1 isoform. The physiological roles of P1-Runx1 and P2-Runx1 proteins were examined in animal models. In contrast to the embryonic lethality caused by a total loss of Runx1 function (*Runx1* ^*Δ/Δ*^ mice), mice lacking only the P1-Runx1 isoform were alive at birth [16, 17]. These mice did not display any apparent defects in early hematopoiesis in the fetal liver. A lack of P1-Runx1 did, however, result in impaired colony forming activity in hematopoietic progenitors in the yolk-sac of 11.5 dpc embryos [16] and affected differentiation of lymphoid tissue inducer (LTi) cells [18]. In adult *Runx1* ^*P1N/P1N*^ mice, basophil development was also impaired [19]. On the other hand, a mouse model with substantially reduced levels of the *P2-Runx1* transcript was also generated by insertion of the neomycin resistant gene (*neo* ^*r*^) upstream of *P2-Runx1* promoter [20]. Mice homozygous for this hypomorphic *Runx1* allele (*Runx1* ^*P2N/P2N*^ mice) were born alive but died a few days after birth [20]. Colony forming activity in embryonic hematopoietic progenitors was more severely impaired in *Runx1* ^*P2N/P2N*^ than in *Runx1* ^*P1N/P1N*^ embryos [16]. Furthermore, when compound mutations were generated between the *Runx1* null mutation (*Runx1* ^*Δ*^) and either the *Runx1* ^*P1N*^ or *Runx1* ^*P2N*^, embryonic lethality and liver anemia were observed in *Runx1* ^*P2N/Δ*^ embryos, but not in *Runx1* ^*P1N/Δ*^ embryos [16]. These observations indicated that the P2-Runx1 isoform is more crucial for definitive hematopoiesis than the P1-Runx1 isoform. Activation of *P2-Runx1* promoter activity generally occurs earlier than that of the *P1-Runx1* promoter [13, 17, 20, 21], and the *P1-Runx1* promoter, which has conserved functional tandem Runx recognition sites [13, 22], appeared later during evolution presumably in ancestral vertebrates [3]. Therefore, the predominant requirement of P2-Runx1 for definitive hematopoiesis is thought to reflect its role in activating the *P1-Runx1* promoter for promoter switching. However, it remains unclear whether P2-Runx1 specific N-terminal sequences, RIPV, are required for early hematopoiesis or not.

In the current study, we generated novel *Runx1* mutant alleles by replacing the translational start AUG (+1) codon of the P2-Runx1 isoform with different STOP codons (UAG or UAA). These models gave unexpected results, showing that the expression of a truncated Runx1 isoform from the *P2-Runx1* promoter alone is sufficient for early hematopoiesis.

## Methods

### Construction of the target vectors and generation of chimera mice

A phage clone containing genomic regions surrounding the* P2-Runx1* promoter was isolated from a phage library purchased from STRATAGEN. A 3′ side short arm fragment was amplified by PCR and was ligated into the pL2Neo2 vector after sequencing, generating a pBlueNeoSA1 vector. A fragment containing the loxP-flanked *neo* ^*r*^ gene and the 3′ short arm was prepared from the pBlueNeoSA1 vector by *XbaI*/*ClaI* digestion and was cloned into the pBS-TK1 vector, generating a pR1pNSATK vector. A 1.9 kb *XhoI*-*SpeI* fragment corresponding to chr16:92,695,251–92,697,182 (mm9) was amplified by PCR and cloned into the pBluescript vector, generating a pR1pEXII vector. A 7.4 kb 5′ long arm fragment corresponding to chr16:92,697,759–92,705,187 was prepared from the phage clone by *NotI* and *SmaI* digestion and was cloned into the pBluescript, generating a pR1pLA1 vector. A 1.9 kb fragment cut out from the pR1pEXII vector by *HincII*/*KpnI* digestion was ligated into the *SmaI*/*KpnI* cleaved pR1PLA1 vector, generating a pR1PLAEXWT vector, in which a 580 bp *SmaI*-*XhoI* region corresponding to chr16:92,697,182–92,697,761 was missing from the 5′ long arm. A fragment containing the TAG replacement mutation was created by overlap PCR and ligated into the *SmaI*/*SpeI*-cleaved pR1pLAEXWT vector, generating a pR1pLAEXMu vector. Finally, a 9.3 kb *NotI*-*SpeI* fragment prepared from the pR1pLAEXMu vector was ligated into the *NotI*/*XbaI-*cleaved pR1pNSATK vector.

To generate the target vector for the *Runx1* ^*P2TAA*^ mutation, overlap PCR was performed with appropriate primers. After sequencing, the 0.8 kb PCR product harboring *SmaI* and *KpnI* at the 5′ and 3′ ends, respectively, and the 7.4 kb *NotI-SmaI* fragment prepared from the pR1pLA1 vector, were ligated using a trimolecular reaction into the *NotI*/*KpnI*-cleaved pBluescript vector, generating a pRIpSmaI vector.

These target vectors (30 μg) were linearized by *ClaI* digestion before transfection into the ES cell line, M1, by electroporation using a GenePulserII (Bio-Rad). After selecting cells with 350 μg/ml G418 (GENETICIN, Gibco) and 2 μM ganciclovir (Wako, 078–04481), individual colonies were subjected to PCR screening with appropriate primers to identify clones that had undergone homologous recombination. ES cell aggregation was performed by the animal facility group at RIKEN IMS.

### Mice

This study was carried out in accordance with guidelines for animal care of the RIKEN Yokohama Campus. Animal experimental protocol was approved by Safety Department at RIKEN Yokohama Campus (Permit Number: 28–017(2)). All mice were maintained in the animal facility at the RIKEN IMS and all animal procedures were in accordance with the institutional guidelines for animal care and were approved by the safety section in RIKEN Yokohama Campus.

### Flow cytometry

Single-cell suspensions were prepared from thymus, spleen, bone marrow and fetal liver, and were stained with the following antibodies, all purchased from BD-Biosciences: CD4 (RM4–5), CD8 (53–6.7), CD49b (DX5), CD117/c-Kit (2B8), IgE (R35–72) and ScaI (E13–161.7). The antibody for FcεRI (MAR-1) was from eBiosciences. Multi-color flow cytometry analysis was performed using a FACS CANTO II (BD-Biosciences), and data were analyzed using FlowJo software (Tree Star).

### Colony forming assay

A colony forming assay on methylcellulose agar was conducted according to the manufacturer’s instructions (Stem Cell Technologies). Single cell suspensions of total fetal liver cells from E11.5 dpc embryos were mixed with MethoCultTM GF M3434 containing SCF, IL3, IL6 and Epo, and plated onto 6-cm dishes, according to the protocols provided. Colony numbers were counted after seven days of culture in a humidified CO_2_ incubator.

### Whole-mount immunostaining

Whole-mount immunostaining of the embryos was performed as previously described [23]. Primary antibodies used were anti-c-Kit (2B8; BD Biosciences) and biotinylated anti-CD31 (MEC13.3; BD Biosciences). The secondary antibody and labeled streptavidin used were goat anti–rat IgG-Alexa Fluor 647 (Invitrogen) and Cy3-streptavidin (Jackson ImmunoResearch Laboratories). Immunostained embryos were mounted in a 1:2 mix of benzyl alcohol and benzyl benzoate (BABB) to increase tissue transparency and analyzed using a confocal microscope (Zeiss LSM 510 Meta, Plan-Neofluar 20×/NA 0.5). Three-dimensional projections were generated from z-stacks using LSM Image Browser (Zeiss).

### Rt-Pcr

Total RNA from embryos and cells was prepared by using TRIzol (ThermoFisher Scientific). After treatment with DNase, 1 μg of total RNA was used to synthesize cDNA using SuperScript™ II Reverse Transcriptase (Invitrogen). Primers used to amplify *P1-* and *P2-Runx2* transcripts were P1-Runx1-F:5′- CTTCAGGAGAGGTGCGTTTTCG -3′, P2-Runx1-F: 5′- CCTCCGGTAGTAATAAAGGCTTC-3′, and Runx1-R: 5′- ATGACGGTGACCAGAGTGCC -3′.. Primers for *Mcpt8* and *bactin* were described previously [19].

### Immunoblotting

Cells were lysed with lysis buffer (20 mM Tris, 150 mM NaCl, 10 mM MgCl2, 0.1% NP-40) containing protease inhibitors (Complete Mini, 11,836,153,001, Roche) and incubated on ice for 20 min. The supernatant was collected after centrifugation at 10,000 rpm for 10 min at 4 °C, mixed with Laemmli sample buffer (Bio-Rad), and subjected to 10% SDS- PAGE followed by transfer to a membrane. Samples were probed with an anit-Runx1 antibody [24] or an anti-β-actin antibody (Sigma, A3853). Immunocomplexes were detected using ECL reagents (Amersham).

### Proteasome inhibitor treatment

One million of CD4^+^ spleen cells were cultured in RPMI1640 media containing10% FBS with or without 10uM MG132 (Calbiochem, Cat# 474791) for one hour before preparation of cell lysate.

## Results

### Characterization of *Runx1*^*P2TAG/P2TAG*^ mutant mice

**Fig. 1:**
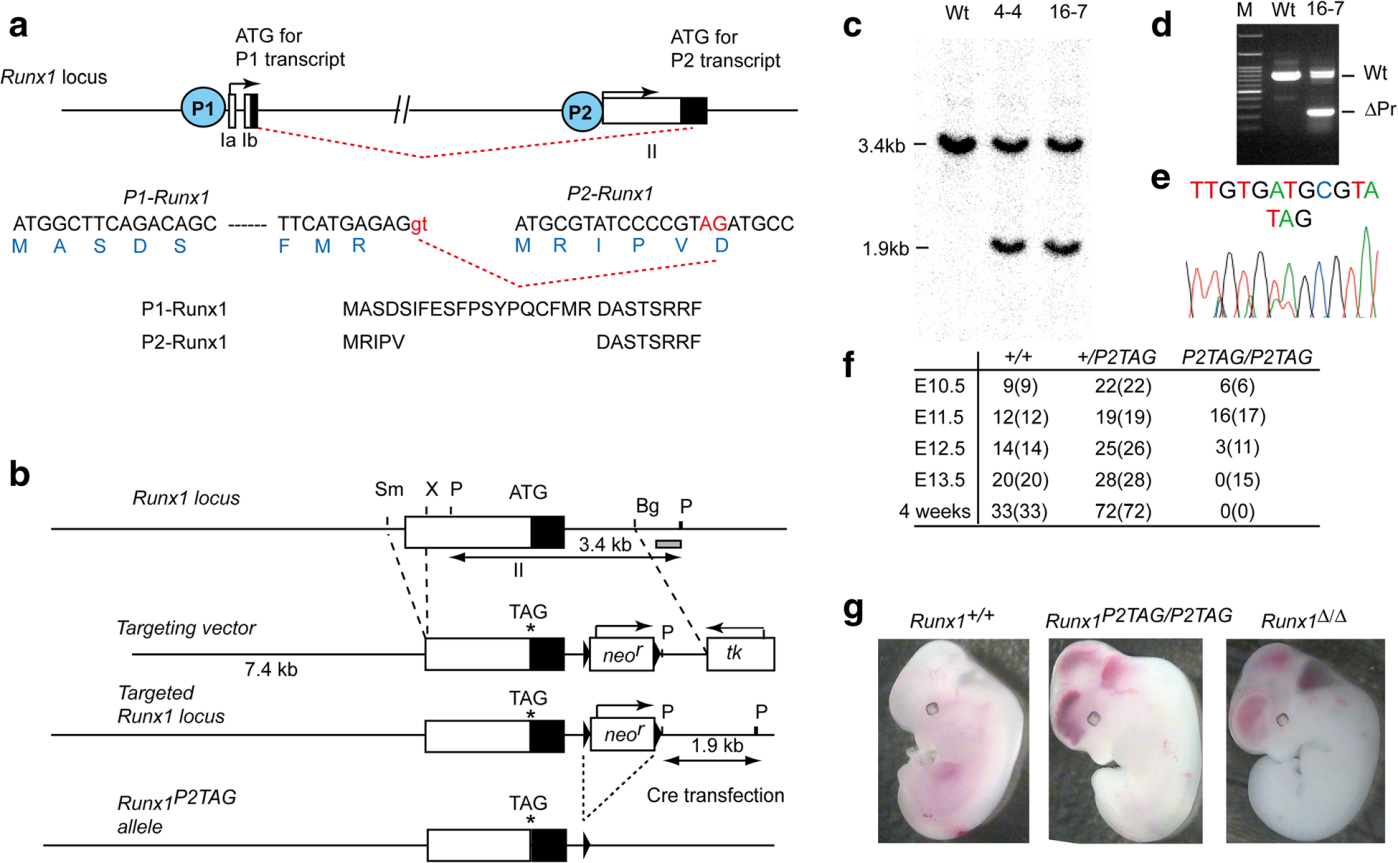
Generation of *Runx1* ^*P2TAG*^ mutant allele.** a** Schematic structure of murine *Runx1* locus. The murine *Runx1* gene is transcribed from distal (P1) and proximal (P2) promoters. Open and closed boxes represent the 5′ untranslated region (UTR) and coding region, respectively. Red dashed lines show the RNA splice junction in the *P1-Runx1* transcript. Nucleotide sequences around the translational start ATG of *P1-* and *P2-Runx1* transcripts are shown. Dashed line in *P1-Runx1* transcript indicates omitted intermediate sequences between the ATG and splice donor signals. Deduced amino acid sequences are shown as a single letter. **b** Schematic representation of the targeting strategy used to generate the *Runx1* ^*P2TAG*^ allele. The targeting vector was designed to delete the *P2-Runx1* promoter between *SmaI* and *XhoI* sites and replace the ATG with TAG, marked with *. Triangles represent loxP sequences. Restriction enzymes shown are; *BglII (Bg)*, *PstI (P)*, *SmaI (Sm)* and *XhoI (X)*. **c** Representative Southern blot of ES clones that underwent homologous recombination. *PstI *digested genome DNA was hybridized with a probe, grey box in **b**. **d** Gel image of DNA-PCR analysis showing incorporation of the P2-promoter deletion in an ES clone. **e** Sequence analysis of the genomic region around the ATG in exon II of the *Runx1* gene. PCR product from ES clone 16–7 was sequenced. **f** Genotyping of offspring obtained by intercrossing between *Runx1* ^*+/P2TAG*^ heterozygous mice. Numbers and those in parenthesis represent live and total embryos, respectively. **g** Representative images of E12.5 dpc *Runx1* ^*+/+*^ and *Runx1* ^*P2TAG/P2TAG*^ embryos. A Runx1-null mutant (*Runx1* ^*Δ/Δ*^) embryo is shown for references

To examine the physiological function of the P2-Runx1 isoform, it is useful to establish a mouse model that specifically lacks this isoform. The extreme 3′ sequences of exon II contains a splice acceptor signal for the *P1-Runx1* transcript, and therefore all sequences after this splice acceptor are shared with the *P1-Runx1* coding sequences (Fig. 1a). We therefore designed a target vector that would replace the translation start codon, AUG, with a stop codon, UAG, to eliminate translation of P2-Runx1 isoform while maintaining intact P1-Runx1 expression (Fig. 1b). We included an additional deletion to remove around 200 bp upstream of the putative transcriptional start site (TSS) of the *P2-Runx1* transcript and part of the 5′UTR of the *P2-Runx1* transcript (Fig. 1B). ES clones that underwent homologous recombination were screened first by PCR and verified by Southern blotting using a probe located at the 3′ end (Fig. 1c). ES clones harboring the P2-promoter deletion were confirmed by PCR (Fig. 1d), while incorporation of the TAG mutation was confirmed by sequencing (Fig. 1e). After removal of the *neo* ^*r*^ gene in ES clones by transient transfection of a Cre expression vector, ES clones were aggregated with eight-cell embryos to generate chimeric mice. These mice were used to establish a mouse line harboring the *Runx1* ^*P2TAG*^ allele. Intercrossing heterozygous mice revealed no homozygous mice (*Runx1* ^*P2TAG//P2TAG*^) in the 4-week-old offspring (Fig. 1f). Analysis of embryos at different developmental stages revealed that *Runx1* ^*P2TAG/P2TAG*^ embryos were alive at 11.5 dpc, but dead at 13.5 dpc (Fig. 1f). In addition, at 12.5 dpc, most of the *Runx1* ^*P2TAG/P2TAG*^ embryos exhibited hemorrhagic regions in the brain and spinal cord (Fig. 1g), as was also seen in Runx1-null mutant (*Runx1* ^*Δ/Δ*^) embryos [7]. Thus, the *Runx1* ^*P2TAG*^ mutation resulted in embryonic lethality around 12.5 dpc, with hemorrhagic characteristics similar to those observed in *Runx1*-null mutants. These findings led us to next examine definitive hematopoiesis in the *Runx1* ^*P2TAG/P2TAG*^ embryos.

**Fig. 2:**
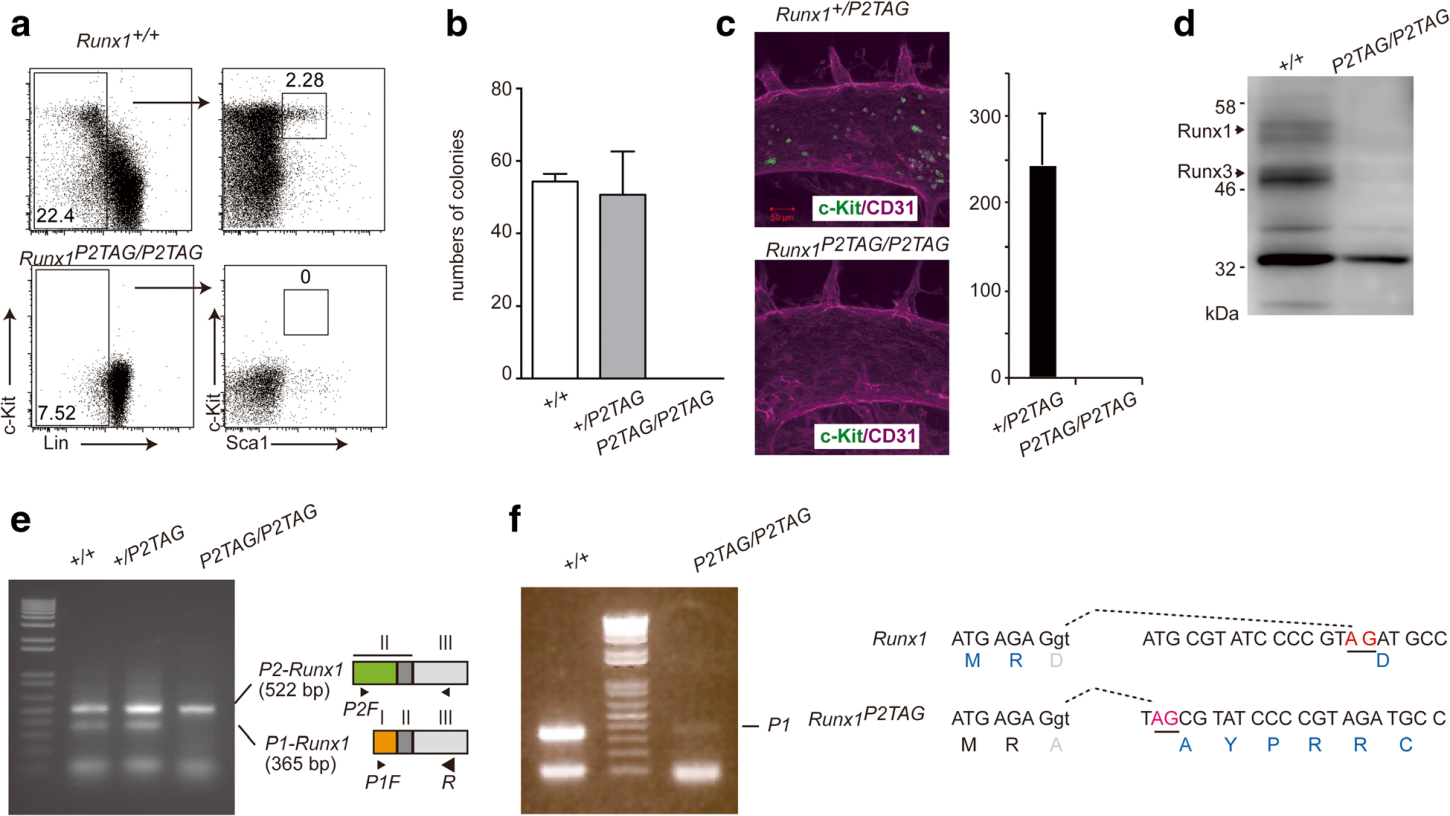
Lack of hematopoietic stem cells (HSC) in *Runx1* ^*P2TAG/P2TAG*^ embryos that harbors an aberrant RNA splice junction in the *P1-Runx1* transcript.** a** Representative dot plots of fetal liver cells from *Runx1* ^*+/+*^ and *Runx1* ^*P2TAG/P2TAG*^ 11.5 dpc embryos. Lin-negative (Lin^−^) cells were analyzed for ScaI and c-Kit expression. **b** Results of three independent colony forming assays of fetal liver cells. No colonies formed from *Runx1* ^*P2TAG/P2TAG*^ cells. **c** Immunohistochemical analysis of c-Kit and CD31 expression in the dorsal aorta of 11.5 dpc embryos. In control *Runx1* ^*+/P2TAG*^ samples, round c-Kit positive cells were seen budding from CD31 positive endothelial cells, whereas those cells were undetectable in *Runx1* ^*P2TAG/P2TAG*^ embryos. Right graph shows a summary of the numbers of c-Kit expressing cells in the dorsal aorta of three embryos. Mean ± SD. **d** Immunoblot showing expression of Runx1 and Runx3 proteins in fetal liver from 11.5 dpc *Runx1* ^*+/+*^ and *Runx1* ^*P2TAG/P2TAG*^ embryos. **e** RT-PCR analysis of *P1-* and *P2-Runx1* transcripts in whole 11.5 dpc *Runx1* ^*+/+*^ *, Runx1* ^*+/P2TAG*^ and *Runx1* ^*P2TAG/P2TAG*^ embryos. Left gel image represents PCR products from a mixture of three primers, whose positions are illustrated on the right. I, II and III represent corresponding exons. **f** RT-PCR analysis focusing on the *P1-Runx1* transcript using two primers, P1F and R. PCR product corresponding to the *P1-Runx1* transcript is indicated. All eight *P1-Runx1* transcripts from the *Runx1* ^*P2TAG/P2TAG*^ embryos examined contained an aberrant splice junction between exon I and II, which resulted from upstream TAG sequences. AG in red font indicates a splice acceptor signal and dashed line indicates a splice junction. Aberrant splicing cause a frame shift and deduced amino acids are shown as a single letter

Flow cytometry analyses of 11.5 dpc embryos showed a decrease in the percentage of lineage-marker-negative (Lin^−^) cells in the liver of *Runx1* ^*P2TAG/P2TAG*^ embryos. In addition, the c-Kit^+^Sca1^+^ subset was undetectable in the Lin^−^ liver population of *Runx1* ^*P2TAG/P2TAG*^ embryos (Fig. 2a), indicating that HSC generation was totally abrogated in these embryos. Consistent with these findings, no hematopoietic colonies arose on methylcellulose dishes comprising *Runx1* ^*P2TAG/P2TAG*^ fetal liver cells, while such colonies were seen in *wild-type* and heterozygous fetal liver cells (Fig. 2b). The lack of c-Kit expressing cells around CD31 expressing endothelial cells in *Runx1* ^*P2TAG/P2TAG1*^ 11.5 dpc embryos was also confirmed by immunohistochemical staining (Fig. 2c). Consistent with lack of HSC, western blot using antibody that recognize both Runx1 and Runx3 proteins [24] failed to detect these proteins in fetal liver cells of *Runx1* ^*P2TAG/P2TAG*^ 11.5 dpc embryo (Fig 2d). We concluded from these observations that the *Runx1* ^*P2TAG*^ allele behaves as functionally *null* allele.

Next, we examined the expression of *P1-* and *P2- Runx1* transcripts using RT-PCR with forward primers specific to each isoform and a common reverse primer (Fig. 2e). In this setting, both *P1-* and *P2-Runx1* transcripts were detected in mRNA prepared from whole *wild-type* and heterozygous 11.5 dpc embryos, but the amount of *P2-Runx1* transcript was higher than *P1-Runx1.* Of note, despite removal of the putative P2-promoter region, the *P2-Runx1* transcript could still be detected in *Runx1* ^*P2TAG/P2TAG*^ embryos at similar levels to control embryos. On the contrary, the *P1-Runx1* transcript was barely detectable in *Runx1* ^*P2TAG/P2TAG*^ embryos (Fig. 2f). Thus, we performed RT-PCR with two primers that specifically amplify it, the *P1-Runx1* transcript was detected also in *Runx1* ^*P2TAG/P2TAG*^ embryos, albeit at very low levels compared to *Runx1* ^*+/+*^ embryos (Fig. 2f). In order to verify whether the PCR products amplified from *Runx1* ^*P2TAG/P2TAG*^ embryos was the real *P1-Runx1* transcript, the PCR product was cloned into a plasmid and sequenced. While sequences at the 5′ end were the same as *P1-Runx1*, we found addition sequences in the middle of all eight clones sequenced. Sequence alignment revealed that the insertion of these additional sequences stemmed from aberrant usage of the inserted TAG as a novel splice acceptor signal (Fig. 2f). Importantly, this aberrant splicing resulted in a frame shift (Fig. 2e), that eventually created a stop codon upstream of the Runt-domain. Thus, the aberrant *P1-Runx1* transcript transcribed from the *Runx1* ^*P2TAG*^ allele generates mostly short truncated peptides that consist of 74 amino acids and lacks the Runt-domain. Sequence analyses of P1-Runx1 transcripts expressed in 11.5dpc *Runx1* ^*+/P2TAG*^ fetal liver found 3 aberrant P1-Runx1 among 31 clones sequence, indicating that non-sense mediated mRNA decay (NMD) of aberrant *P1-Runx1 mRNA* is likely to be involved in reduction of *P1-Runx1* mRNA in *Runx1* ^*P2TAG/P2TAG*^ embryos*.* With these observations, we concluded that the phenocopying of *Runx1* ^*P2TAG/P2TAG*^ and *Runx1* ^*Δ/Δ*^ embryos was likely caused by a combined loss of the P1-Runx1 isoform and attenuated or loss of P2-Runx1 isoform expression.

### Characterization of *Runx1*^*P2TAA/P2TAA*^ mutant mice

**Fig. 3:**
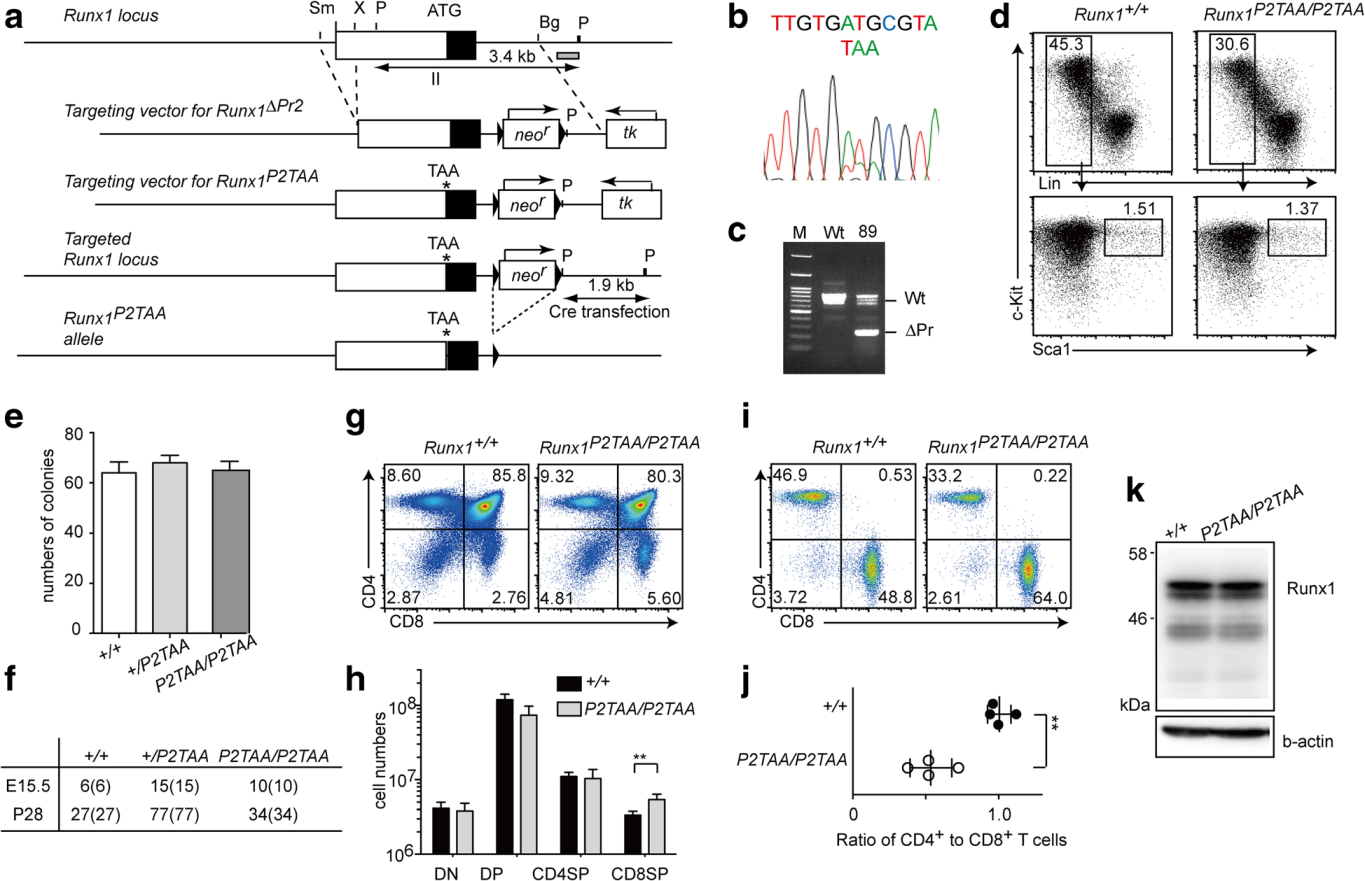
Generation of *Runx1* ^*P2TAA*^ mutant mice. **a** Schematic representation of the targeting strategy used to generate the *Runx1* ^*P2TAA*^ allele. A targeting vector was designed to replace ATG with TAA, marked with *. Open and closed boxes represent the 5′ untranslated region (UTR) and coding region in exon II, respectively. Neomycin resistance (*neo* ^*r*^) and thymidine kinase (*tk)* genes were used for positive and negative selection, respectively. Triangles represent loxP sequences. **b** Sequence analysis of the genomic region around the ATG in exon II of the *Runx1* gene in an ES clone showing replacement of ATG with TAA. **c** Gel image of DNA-PCR analysis showing incorporation of the P2-promoter deletion in an ES clone. **d** One representative dot plots of at least three individual experiments showing ScaI and c-Kit expression in Lin-negative (Lin^−^) fetal liver cells from 11.5 dpc *Runx1* ^*+/+*^ and *Runx1* ^*P2TAA/P2TAA*^ embryos. **e** One representative of two colony forming assay of fetal liver cells from mice of indicated genotypes. **f** Genotyping of offspring obtained by intercrossing *Runx1* ^*+/P2TAA*^ heterozygous mice at 15.5 dpc and four weeks (P28). Numbers and those in parenthesis represent live and total mice, respectively. **g** Dot plots showing CD4 and CD8 expression in total thymocytes of three week-old *Runx1* ^*+/+*^ and *Runx1* ^*P2TAA/P2TAA*^ mice. **h** Graph showing absolute numbers of thymocyte subsets. DN: CD4^−^CD8^−^ double negative. DP: CD4^+^CD8^+^ double positive. CD4SP: CD4^+^CD8^−^ single positive. CD8SP: CD4^−^CD8^+^ single positive. Mean ± SD. ** *P* < 0.01. **i** Dot plots showing CD4 and CD8 expression in splenic T cells of three week-old *Runx1* ^*+/+*^ and *Runx1* ^*P2TAA/P2TAA*^ mice. **j** Graph showing CD4^+^ to CD8^+^ T cells in lymph nodes. Mean ± SD. ** *P* < 0.01. **k** Immunoblot showing expression of Runx1 protein in total thymocytes from three week-old *Runx1* ^*+/+*^ and *Runx1* ^*P2TAA/P2TAA*^ mice. One representative image of two experiments

Having shown that the replacement of ATG with TAG created an aberrant splice acceptor site, we designed a second target vector to replace the ATG with TAA, another stop codon, hereafter referred to as a *Runx1* ^*P2TAA*^ mutation (Fig. 3a). Since we wanted to examine the effect of *P2-Runx1* promoter deletion and the *Runx1* ^*P2TAA*^ mutation separately, we constructed two vectors, which would target the *Runx1* ^*P2TAA*^ mutation or *P2-Runx1* promoter deletion (Fig. 3a), in the second gene targeting. We isolated ES clones harboring the *Runx1* ^*P2TAA*^ mutation (Fig. 3b), from which we established a mutant mouse line. However, although we isolated ES clones harboring the *P2-Runx1* promoter deletion (Fig. 3c) and generated chimeric mice, the *P2-Runx1* promoter deletion was not transmitted to the next generation. Therefore we could not generate the mouse line harboring the *P2-Runx1* promoter deletion alone.

Analysis of liver cells from 11.5 dpc embryos obtained from intercrossing *Runx1* ^*+/P2TAA*^ heterozygotes detected Lin^−^c-Kit^+^ScaI^+^ cells in the *Runx1* ^*P2TAA/P2TAA*^ embryos to the same extent as in control embryos (Fig. 3d). Colony forming activity in fetal liver cells was also comparable between *Runx1* ^*+/+*^ and *Runx1* ^*P2TAA/P2TAA*^ embryos (Fig. 3e). Thus, early definitive hematopoiesis during embryogenesis was not affected by the *Runx1* ^*P2TAA*^ mutation. In addition, we observed that homozygous *Runx1* ^*P2TAA/P2TAA*^ mice were alive at birth and grew normally (Fig. 3f).

T lymphocyte development has been shown to be sensitive to *Runx1* dosage. For example, the ratio of CD4-helper to CD8-cytotoxic T subsets in peripheral lymphoid tissues was shown to be reverted in heterozygous *Runx1* ^*+/Δ*^ mice [25]. We therefore examined T cell development in 3–5 week-old *Runx1* ^*P2TAA/P2TAA*^ mice. There were no significant changes in total thymocyte number, but the percentage and absolute number of CD8SP thymocyte subsets was slightly increased (Fig. 3g and h). The ratio of CD4^+^ to CD8^+^ T cells was also reverted in peripheral lymphoid tissues such as the spleen in *Runx1* ^*P2TAA/P2TAA*^ mice (Fig. 3i and j). This finding suggests that Runx1 activity is compromised in *Runx1* ^*P2TAA/P2TAA*^ mice. However, the total amount of Runx1 protein, as analyzed by immunoblotting, was similar between *Runx1* ^*+/+*^ and *Runx1* ^*P2TAA/P2TAA*^ thymocytes (Fig. 3k), presumably because the P1-Runx1 isoform is expressed in those cells.

### Generation of *Runx1*^*ΔP1:P2TAA*^ Mouse line

Previous reports have suggested the existence of truncated Runx2 and Runx3 proteins due to a non-canonical AUG codon downstream of the canonical AUG (+1) [26, 27]. Thus, it is possible that the *Runx1* ^*P2TAA*^ allele generates a truncated Runx1 protein from the *P2-Runx1* transcript via a non-canonical AUG codon, which would be produced in addition to the normal P1-Runx1 isoform. However, it was not possible to analyze whether and what types of truncated Runx1 protein were expressed from the *Runx1* ^*P2TAA*^ allele, because there are no available antibodies that will distinguish between these two proteins. Theoretically, it is possible that a similar truncated P2-Runx1 protein could be generated from the *Runx1* ^*P2TAG*^ allele. However, such a putative truncated protein would not be sufficient to support early embryogenesis in the absence of the P1-Runx1 isoform. To overcome these limitations, and test whether elimination of the P1-Runx1 isoform over the *Runx1* ^*P2TAA*^ allele recapitulates the *Runx1* ^*P2TAG*^ phenotype, we generated another mutant *Runx1* allele, referred to as *Runx1* ^*ΔP1:P2TAA*^, by targeting the *Runx1* ^*P2TAA*^ mutation to the *Runx1* ^*ΔP1*^ allele (Additional file 1).

**Fig. 4:**
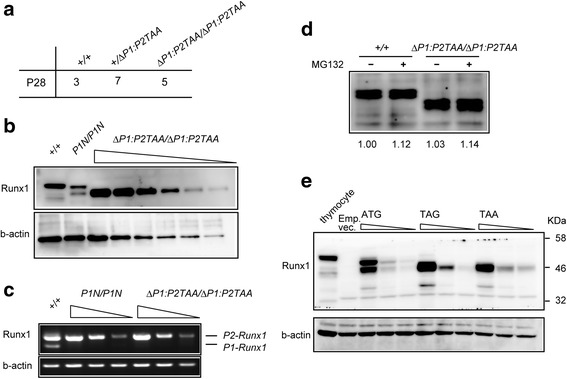
Expression of truncated Runx1 protein from the *Runx1* ^*ΔP1:P2TAA*^ allele*.* **a** Genotyping of offspring obtained by intercrossing *Runx1* ^*ΔP1:P2TAA*^ heterozygous mice at four weeks. **b** Cell lysates prepared from total thymocytes of adult mice of indicated genotypes were immunoblotted with an anti-Runx1 antibody. Serial dilutions, each reduced by half, were prepared from cell lysates from *Runx1* ^*ΔP1:P2TAA/ΔP1:P2TAA*^ mice. The membrane was re-blotted with an anti b-actin antibody as an internal control. One representative image of two experiments. **c** Semi-quantitative RT-PCR analysis showing similar amounts of *P2-Runx1* transcript between *Runx1* ^*P1N/P1N*^ and *Runx1* ^*ΔP1:P2TAA/ΔP1:P2TAA*^ thymocytes. **d** Effect of proteasome inhibitor, MG132, on the amount of Runx1 proteins. CD4^+^ T cells prepared from *Runx1* ^*+/+*^ and *Runx1* ^*ΔP1:P2TAA/ΔP1:P2TAA*^ mice were treated with 10 μM MG132 proteasome inhibitor for one hour. Numbers at the bottom indicate relative expression level to that of *Runx1* ^*+/+*^ cells without MG132 treatment. **e** Expression of Runx1 protein from three expression vectors harboring different sequences around the translation start site, ATG, TAG and TAA, on P2-Runx1 cDNA. Different amounts (1.0, 0.5, and 0.25 μg) of these vectors were transfected into 293 T cells. Forty-eight hours after transfection, cell lysates were immunoblotted with an anti-Runx1 antibody. Empty vector (Emp.Vec.) and thymocyte lysates were included as references

Unexpectedly, homozygous *Runx1* ^*ΔP1:P2TAA/ ΔP1:P2TAA*^ mice were born alive and grew normally (Fig. 4a). Thus, not only is the *Runx1* ^*ΔP1:P2TAA*^ allele completely different from the *Runx1* ^*P2TAG*^ allele, but the truncated P2-Runx1 protein generated from the *Runx1* ^*ΔP1:P2TAA*^ allele also provides sufficient Runx1 function for early mouse development. We then examined Runx1 protein expression in total thymocytes by immunoblotting. In *Runx1* ^*P1N/P1N*^ thymocytes that lack exon I of the *Runx1* gene, two different sizes of Runx1 protein were detected (Fig. 4b), indicating that the *P2-Runx1* transcript produced two isoform protein. Interestingly, the protein with the heavier molecular weight was not detected in *Runx1* ^*ΔP1:P2TAA/ ΔP1:P2TAA*^ cells (Fig. 4b). Given that the major difference between the *Runx1* ^*P1N*^ and *Runx1* ^*ΔP1:P2TAA*^ alleles is the ATG to TAA replacement in exon II, the P2-Runx1 isoform detected at the upper position in *Runx1* ^*P1N/P1N*^ cells should be P2-Runx1 protein translated from canonical AUG (+1), whereas the protein detected at the lower position was likely translated from the non-canonical downstream AUG. The truncated P2-Runx1 protein detected in *Runx1* ^*ΔP1:P2TAA/ ΔP1:P2TAA*^ cells was of a similar size to the one detected at the lower position in *Runx1* ^*P1N/P1N*^ cells, suggesting that both cells utilize the same non-canonical downstream AUG. There are AUGs at positions +73 and +151, as well as others further downstream, which are in frame with the canonical AUG (+1). Functional compensation by the truncated P2-Runx1 isoform disfavors the option to use AUG (+151), because the truncated protein would lack the first two amino acids of the Runt-domain. In addition, the sequence around AUG (+73) was predicted to be closer to Kozak sequences than AUG (+151) according to the ATGpr program [28]. We therefore propose that the position of the non-canonical downstream AUG is at +73. Interestingly, we found that the amount of truncated P2-Runx1 isoform in *Runx1* ^*ΔP1:P2TAA/ ΔP1:P2TAA*^ thymocytes was higher than that of Runx1 protein in *Runx1* ^*+/+*^ and *Runx1* ^*P1N/P1N*^ cells (Fig. 4b). Semi-quantitative RT-PCR showed no clear differences in the amount of *P2-Runx1* transcript between the *Runx1* ^*P1N*^ and *Runx1* ^*ΔP1:P2TAA*^ alleles (Fig. 4c), suggesting that post-transcriptional mechanisms may regulate the stability of the truncated P2-Runx1 protein. We then tested whether proteasome mediated degradation is involved in stability of truncated Runx1 protein by using proteasome inhibitor, MG132, and observed that amount both wild-type and truncated Runx1 proteins were slightly increased by treatment of CD4^+^ T cells with MG132 (Fig. 4d).

Next, we examined whether translation of Runx1 protein from the non-canonical AUG occurs in non-hematopoietic cells and from an artificial *P2-Runx1* transcript derived from a plasmid, which has a short (10 bp) 5′UTR region. Three expression vectors were constructed by inserting a cDNA fragment encoding either wild-type *P2-Runx1* or mutant cDNA fragments harboring a TAG or TAA replacement at the canonical ATG (+1) into a pcDNA3 expression vector. These were then transfected into non-hematopoietic 293 T cells. As observed in thymocytes, two Runx1 isoforms were produced from the wild-type *P2-Runx1* construct, while both the TAG and TAA mutant constructs generated only the smaller Runx1 isoform (Fig. 4e). These results suggest that use of the non-canonical AUG is not specific to thymocytes, and that it is not affected by the TAG mutation.

### Restored basophil development in *Runx1*^*ΔP1/P2TAA*^mice

**Fig. 5:**
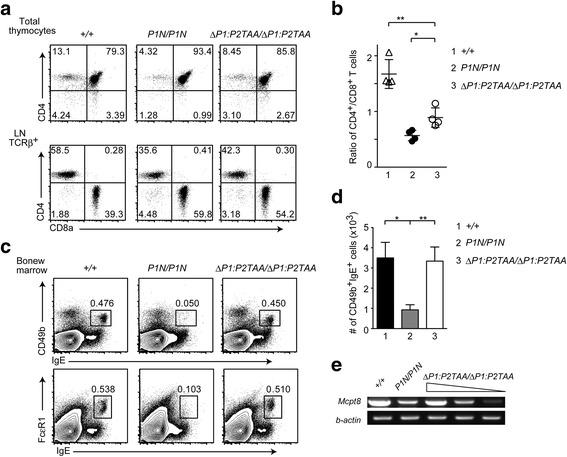
Restored basophil development in *Runx1* ^*ΔP1:P2TAA /ΔP1:P2TAA*^ mice. **a** Dot plots showing CD4/CD8 expression in total thymocytes (upper) and lymph node T cells (lower) of 4 to 6 week-old *Runx1* ^*P1N/P1N*^ and *Runx1* ^*ΔP1:P2TAA: ΔP1:P2TAA*^ mice. **b** Graph showing CD4^+^ T cells to CD8^+^ T cells in lymph nodes of *Runx1* ^*+/+*^ (lane 1), *Runx1* ^*P1N/P1N*^ (lane 2) and *Runx1* ^*ΔP1:P2TAA: ΔP1:P2TAA*^ mice (lane 3) mice. Mean ± SD. * *P* < 0.05. ** *P* < 0.01. **c** Flow cytometry analysis of basophil differentiation. Dot plots showing expression of IgE, CD49b and FcεRI in bone marrow cells from *Runx1* ^*+/+*^, *Runx1* ^*P1N/P1N*^ and *Runx1* ^*ΔP1:P2TAA: ΔP1:P2TAA*^ mice. **d** Graphs showing numbers of CD49b^+^IgE^+^ cells in bone marrow from *Runx1* ^*+/+*^ (lane 1), *Runx1* ^*P1N/P1N*^ (lane 2) and *Runx1* ^*ΔP1:P2TAA: ΔP1:P2TAA*^ mice (lane 3) mice. Mean ± SD. * *P* < 0.05, **P < 0.01. **e** Semi-quantitative RT-PCR analysis of *Mcpt8* transcript, a molecular marker of basophils, in bone marrow cells from mice of indicated genotypes. One representative of two experiments

Only the truncated Runx1 protein lacking unique N-terminal sequences specific to either P1-Runx1 or P2-Runx1 were expressed in *Runx1* ^*ΔP1:P2TAA/ ΔP1:P2TAA*^ mice, but we did not observe any major defects in early hematopoiesis or thymocyte development (Fig. 5a). However, the reverted CD4/CD8 ratio observed in peripheral lymphoid tissues of *Runx1* ^*P1N/P1N*^ mice was restored to some extent in the *Runx1* ^*ΔP1:P2TAA/ ΔP1:P2TAA*^ mice, although there was still some skewing towards the CD8-lineage (Fig. 5a and b). These observations indicate that the increased amount of truncated P2-Runx1 in *Runx1* ^*ΔP1:P2TAA/ ΔP1:P2TAA*^ cells compared to *Runx1* ^*P1N/P1N*^ cells has the potential to restore the defect in hematopoietic cell differentiation caused by loss of the P1-Runx1 isoform. Since we had previously observed that basophil development was impaired in *Runx1* ^*P1N/P1N*^ mice [19], we next examined whether basophil development remained impaired or was restored in *Runx1* ^*ΔP1:P2TAA/ ΔP1:P2TAA*^ mice. Basophils can be detected in the bone marrow as a CD49b^+^IgE^+^ or FcεRI^+^ IgE^+^ population. As previously reported, these cell populations were reduced in *Runx1* ^*P1N/P1N*^ mice (Fig. 5c and d). On the contrary, the percentage and number of basophils in the bone marrow of *Runx1* ^*ΔP1:P2TAA/ ΔP1:P2TAA*^ mice were restored to levels comparable to control mice (Fig. 5c and d). Furthermore, levels of *Mcpt8* gene expression, a molecular marker of basophils, were also restored in the bone marrow cells of *Runx1* ^*ΔP1:P2TAA/ ΔP1:P2TAA*^ mice (Fig. 5e). This provides further support that the truncated P2-Runx1 isoform derived from the *Runx1* ^*ΔP1:P2TAA*^ allele, compared to total P2-Runx1 protein that consists of both wild-type and truncated P2-Runx1 protein from the *Runx1* ^*P1N*^ allele, can compensate for P1-Runx1 function more efficiently during basophil development.

## Discussion

In the present study, we generated three novel *Runx1* mutant mouse models, *Runx1* ^*P2TAA/P2TAA*^, *Runx1* ^*P2TAG/P2TAG*^ and *Runx1* ^*ΔP1:P2TAA/ ΔP1:P2TAA*^, and addressed the function of the *P2-Runx1* transcript during hematopoiesis. Unexpectedly, a mutation that replaced the canonical translational AUG (+1) codon with UAG, named the *Runx1* ^*P2TAG*^ allele, created an aberrant splice acceptor site. Joining of the *P1-Runx1* transcript using this aberrant splice acceptor not only occurred more efficiently than that from the canonical splice acceptor, but also resulted in a frame shift. The *P1-Runx1* transcript from the *Runx1* ^*P2TAG*^ allele thus could generate mostly short peptides that lacked the Runt-domain. The mechanism by which splice donor/acceptor sites are selected and utilized remains unclear, therefore it is difficult to identify the reason for preferential use of the aberrant splice acceptor created by the TAG replacement. In the *Runx1* ^*P2TAG*^ allele, we deleted a 580 bp genomic region that included a 200 bp region upstream of the transcriptional start site (TSS), which corresponds to a putative *P2-Runx1* promoter region. However, we still observed a P*2-Runx1* transcript in *Runx1* ^*P2TAG/P2TAG*^ embryos at 11.5 dpc, suggesting the presence of another promoter(s) that drives transcription from a site close to exon II. Indeed, a recent FANTOM5 database comprising numerous TSS from Cap Analysis of Gene Expression (CAGE) identified a cryptic TSS that mapped to a region 220 bp downstream of the canonical TSS for the *P2-Runx1* transcript (Additional file 2). Although this second TSS was also deleted in the *Runx1* ^*P2TAG*^ allele, it raises the possibly that additional promoter(s) could drive *P2-Runx1* transcription. It has been proposed that shadow enhancers become active when the primary enhancer becomes non-functional, in order to ensure robustness [29]. Thus, a similar backup system may be in place to activate a hidden promoter, thus ensuring transcription of *P2-Runx1* from the *Runx1* ^*P2TAG*^ allele.

Production of the same truncated Runx1 protein from expression vectors harboring either a TAG or TAA replacement suggests that a truncated Runx1 protein may be produced from the *P2-Runx1* transcript transcribed from *the Runx1* ^*P2TAG*^ allele. However, in contrast to *Runx1* ^*ΔP1:P2TAA/ ΔP1:P2TAA*^ mice, which survive beyond birth, *Runx1* ^*P2TAG/P2TAG*^ embryos die at 12.5 dpc, as is also seen in *Runx1* ^*Δ/Δ*^ embryos. Therefore, even though a truncated P2-Runx1 isoform is produced from the *P2-Runx1* transcript driven by a putative shadow promoter in *Runx1* ^*P2TAG/P2TAG*^ mice, the amount is unlikely to be sufficient to support mouse embryogenesis. This is supported by the previous observation that embryos expressing a compound mutation of the *Runx1* ^*P2Neo*^ with the *Runx1* ^*P1N*^ allele (Runx1 ^*P2Neo /P1N*^embryos) also died at 12.5 dpc. Based on the earlier expression of the *P2-Runx1* transcript and the presence of conserved functional Runx sites within the *P1-Runx1* promoter [13, 22], it has been proposed that the P2-Runx1 isoform may activate the P1-Runx1 promoter. It was also shown that mice lacking the IRES sequences in the *P2-Runx1* transcript died around 14.5 dpc [30]. Thus, expression of P2-Runx1 above a certain amount is necessary to support mouse embryogenesis until the *P1-Runx1* promoter is fully activated and able to produce enough P1-Runx1 isoform to compensate for low levels of the P2-Runx1 isoform.

Even though *Runx1* ^*ΔP1:P2TAA/ ΔP1:P2TAA*^ mice produced only the truncated P2-Runx1 protein, whose translation would start from the AUG (+73), these mice grew normally and did not display any major defects in hematopoiesis. These findings indicate that N-terminal sequences specific to either the P1-Runx1 or P2-Runx1 isoform are dispensable for most Runx1 functions. Rather, an increase in the amount of truncated P2-Runx1 protein, which is presumably regulated at the post-transcriptional level, suggests that the N-terminal amino acid sequences (Arg2 to Lys24) that are lost in the truncated P2-Runx1 isoform function as a negative regulatory domain for protein stability. A previous study showed that the N-terminal region of the human Runx1 protein contains an auto-inhibitory domain that inhibits hetero-dimerization with Cbfβ/PEBP2β [31]. Dimerization with Cbfβ/PEBP2β prevents ubiquitin-mediated degradation and is therefore important for stability of the Runx1 protein [32]. The truncated Runx1 protein that is translated from the non-canonical AUG (+73) lacks a lysine residue (Lys24), and it is possible that this Lys24 serves as an ubiquitination site, since its replacement by arginine (Lys24Arg) was shown to increase protein stability [32]. Thus, escaping protein degradation, through either enhanced dimerization or reduced ubiquitination due to lack of the N-terminal, is at least partly responsible for the increase in truncated P2-Runx1 protein. In this case, the fact that more of the canonical P2-Runx1 isoform than the truncated P2-Runx1 isoform is produced in thymocytes from *Runx1* ^*P1N/P1N*^ mice suggests that the efficacy of translation is higher from the canonical AUG (+1).

Finally, our results revealed that the truncated Runx1 protein produced from the *Runx1* ^*ΔP1:P2TAA*^ allele was able to restore the defect in basophil development due to absence of the P1-Runx1 isoform. The enhanced function of the truncated P2-Runx1 isoform over the canonical P2-Runx1 isoform is likely to stem mainly from the higher amount, although other possibilities, such as changes in affinity for partner proteins due to conformational changes, cannot be formally excluded. On the other hand, the reverted CD4/CD8 T cell ratio was not completely restored by the truncated P2-Runx1 isoform. A previous study showed that overexpression of the P2-Runx1 isoform from a transgene caused a skew towards CD8-lineage differentiation [33]. Along with a CD8-skewing in the presence of P1-Runx1 in the *Runx1* ^*P2TAA/P2TAA*^ mice, an increase in the amount of truncated P2-Runx1 could have a similar effect on T lymphocyte development. Given the lack of P2-Runx1-specific N-terminal sequences in the truncated protein, this effect would not be specific to the canonical P2-Runx1 isoform. A similar CD8-skew in differentiation was observed with a half-dosage of the *Runx1* gene [25], suggesting that tight regulation of the amount of Runx1 protein is essential for appropriate T lymphocyte development. It is interesting that the canonical AUG (+1) is predominately used to generate the canonical P2-Runx1 isoform, which is more unstable than the truncated P2-Runx1 isoform. Together with evolutionary conservation of the N-terminal sequences in P2-Runx1 proteins, any potential negative regulatory function endowed on the N-terminal sequences of the Runx1 protein could have a physiological role in adjusting the amount of Runx1 protein to appropriate levels.

## Conclusions

Generation of isoform proteins by differential usage of alternative promoter or RNA splicing contributes to increase functional diversification of the gene product. The findings by our genetic approaches modulating the translation start codon on the *P2-Runx2* transcript, which were combined with loss of the P1-Runx1 isoform, unraveled not only that unique N-terminal sequences specific to P1-Runx1 or P2-Runx2 are dispensable for Runx1 function supporting embryogenesis and early hematopoiesis, but also that the N-terminal sequences in the P2-Runx1 isoform have a role in fine-tuning the Runx1 protein level through de-stabilizing P2-Runx1 isoform.

## Additional files

Additional file 1: Strategy used to generate Runx1DP1:P2TAA mutant allele by sequential gene targeting. Schematic representation of the targeting strategy used to generate the Runx1DP1:P2TAA allele. Open and closed boxes represent the 5′ untranslated region (UTR) and coding region in exon I and II, respectively. The neor and tk indicate neomycin resistance and thymidine kinase genes, respectively. Triangles represent loxP sequences. To select ES cells with G418 after transfection of the target vector for the *Runx1* ^*P2TAA*^ mutation, the neor gene was removed from ES clones harboring the *Runx1* ^*+/P1N*^ genotype, thus generating ES clones harboring the *Runx1* ^*+/*Δ*P1*^ genotype. Cells were transfected with the target vector for the *Runx1* ^*P2TAA*^ mutation and clones that underwent homologous recombination were isolated. To screen for whether the Runx1 or Runx1DP1 allele was targeted to the *Runx1* ^*P2TAA*^ mutation, ES clones were transduced with a retroviral vector encoding Cre recombinase and screened by PCR for an inverted recombination event between loxP sequences in opposite directions. ES clones harboring the *Runx1* ^*+/*Δ*P1:P2TAAN*^ genotype were isolated. Primers are indicated as red arrowheads. Gel image on the right shows detection of inverted recombination in clones 3–8 and 9–8. The *neo* ^*r*^ gene was removed by transient transfection of Cre recombinase to isolate ES clones harboring the *Runx1* ^*+/ΔP1:P2TAA*^ genotype. (PDF 209 kb)

Additional file 2: Detection of a cryptic TSS that mapped to a region 220 bp downstream of the canonical TSS in the public FANTOM5 database. Image of FANTOM5 web browser showing canonical and cryptic transcriptional start site (TSS), which are marked with arrow heads, for P2-Runx1 transcript. Red line indicates a genomic region that was deleted in the Runx1P2TAG allele. Numbers represent nucleotide positons according to mm9 reference. (PDF 84 kb)

## Abbreviations

AML: Acute myeloid leukemia
CAGE: Cap Analysis of Gene Expression
dpc: Days post coitum
ES cells: Embryonic stem cells
FcεRI: Fc epsilon receptor I
HSC: Hematopoietic stem cell
Mcpt8: Mast cell protease 8
RT-PCR: Reverse transcription-polymerase chain reaction
Runx: Runt-related transcription factors
TSS: Transcriptional start site
