# Supplementary material for: Unique N-terminal sequences in two Runx1 isoforms are dispensable for Runx1 function
Source: BMC Dev Biol. 2017 Oct 18;17:14. doi: 10.1186/s12861-017-0156-y (PMC5648507; doi:10.1186/s12861-017-0156-y)
Supplement: Supplementary file 1 — Strategy used to generate Runx1DP1:P2TAA mutant allele by sequential gene targeting. Schematic representation of the targeting strategy used to generate the Runx1DP1:P2TAA allele. Open and closed boxes represent the 5′ untranslated region (UTR) and coding region in exon I and II, respectively. The neor and tk indicate neomycin resistance and thymidine kinase genes, respectively. Triangles represent loxP sequences. To select ES cells with G418 after transfection of the target vector for the Runx1 P2TAA mutation, the neor gene was removed from ES clones harboring the Runx1 +/P1N genotype, thus generating ES clones harboring the Runx1 +/ΔP1 genotype. Cells were transfected with the target vector for the Runx1 P2TAA mutation and clones that underwent homologous recombination were isolated. To screen for whether the Runx1 or Runx1DP1 allele was targeted to the Runx1 P2TAA mutation, ES clones were transduced with a retroviral vector encoding Cre recombinase and screened by PCR for an inverted recombination event between loxP sequences in opposite directions. ES clones harboring the Runx1 +/ΔP1:P2TAAN genotype were isolated. Primers are indicated as red arrowheads. Gel image on the right shows detection of inverted recombination in clones 3–8 and 9–8. The neo r gene was removed by transient transfection of Cre recombinase to isolate ES clones harboring the Runx1 +/ΔP1:P2TAA genotype. (PDF 209 kb) [file 12861_2017_156_MOESM1_ESM.pdf]

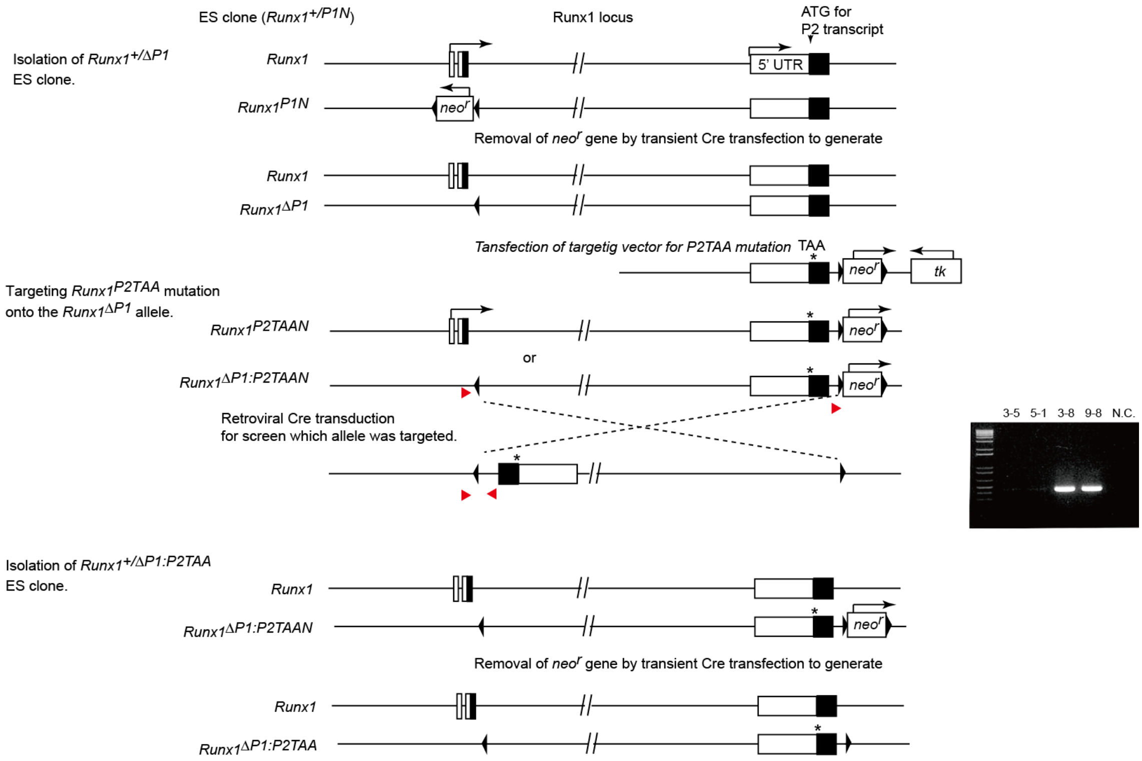


**Additional file1. Strategy used to generate *Runx1*$^{\Delta P1:P2TAA}$ mutant allele by sequential gene targeting.** Schematic representation of the targeting strategy used to generate the *Runx1*$^{\Delta P1:P2TAA}$ allele. Open and closed boxes represent the 5′ untranslated region (UTR) and coding region in exon I and II, respectively. The *neo*$^{r}$ and *tk* indicate neomycin resistance and thymidine kinase genes, respectively. Triangles represent loxP sequences. To select ES cells with G418 after transfection of the target vector for the *Runx1*$^{P2TAA}$ mutation, the *neo*$^{r}$ gene was removed from ES clones harboring the *Runx1*$^{+/P1N}$ genotype, thus generating ES clones harboring the *Runx1*$^{+/\Delta P1}$ genotype. Cells were transfected with the target vector for the *Runx1*$^{P2TAA}$ mutation and clones that underwent homologous recombination were isolated. To screen for whether the *Runx1* or *Runx1*$^{\Delta P1}$ allele was targeted to the *Runx1*$^{P2TAA}$ mutation, ES clones were transduced with a retroviral vector encoding Cre recombinase and screened by PCR for an inverted recombination event between loxP sequences in opposite directions. ES clones harboring the *Runx1*$^{+/\Delta P1:P2TAAN}$ genotype were isolated. Primers are indicated as red arrowheads. Gel image on the right shows detection of inverted recombination in clones 3-8 and 9-8. The *neo*$^{r}$ gene was removed by transient transfection of Cre recombinase to isolate ES clones harboring the *Runx1*$^{+/\Delta P1:P2TAA}$ genotype.
