# Supplementary material for: Unique N-terminal sequences in two Runx1 isoforms are dispensable for Runx1 function
Source: BMC Dev Biol. 2017 Oct 18;17:14. doi: 10.1186/s12861-017-0156-y (PMC5648507; doi:10.1186/s12861-017-0156-y)
Supplement: Supplementary file 2 — Detection of a cryptic TSS that mapped to a region 220 bp downstream of the canonical TSS in the public FANTOM5 database. Image of FANTOM5 web browser showing canonical and cryptic transcriptional start site (TSS), which are marked with arrow heads, for P2-Runx1 transcript. Red line indicates a genomic region that was deleted in the Runx1P2TAG allele. Numbers represent nucleotide positons according to mm9 reference. (PDF 84 kb) [file 12861_2017_156_MOESM2_ESM.pdf]

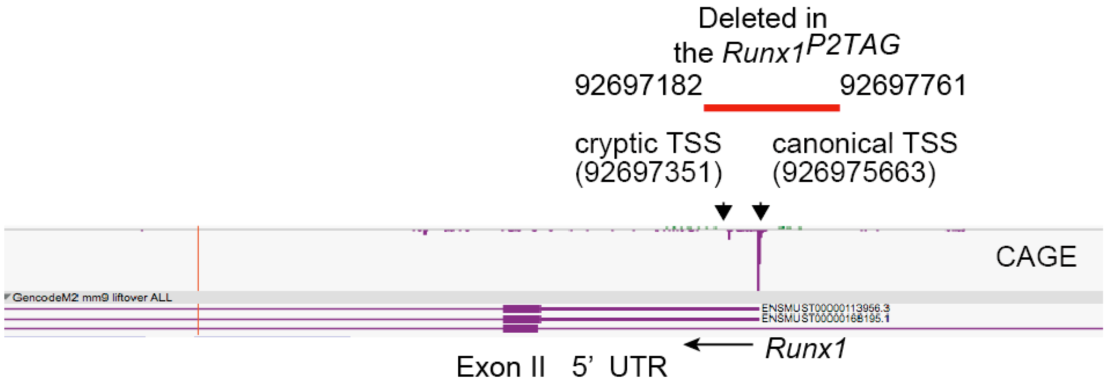

Additional file 2. Detection of a cryptic TSS that mapped to a region 220 bp downstream of the canonical TSS in the public FANTOM5 database. Image of FANTOM5 web browser showing canonical and cryptic transcriptional start site (TSS), which are marked with arrow heads, for *P2-Runx1* transcript. Red line indicates a genomic region that was deleted in the *Runx1*^{P2TAG} allele. Numbers represent nucleotide positons according to mm9 reference.
